# Monitoring of nutrient limitation in growing *E. coli*: a mathematical model of a ppGpp-based biosensor

**DOI:** 10.1186/s12918-017-0490-5

**Published:** 2017-11-21

**Authors:** Alexandra Pokhilko

**Affiliations:** Institute of Molecular, Cell and Systems Biology, University of Glasgow, Glasgow, Scotland UK

**Keywords:** Mathematical modelling, *E. coli*, ppGpp, Biosensor

## Abstract

**Background:**

*E. coli* can be used as bacterial cell factories for production of biofuels and other useful compounds. The efficient production of the desired products requires careful monitoring of growth conditions and the optimization of metabolic fluxes. To avoid nutrient depletion and maximize product yields we suggest using a natural mechanism for sensing nutrient limitation, related to biosynthesis of an intracellular messenger - guanosine tetraphosphate (ppGpp).

**Results:**

We propose a design for a biosensor, which monitors changes in the intracellular concentration of ppGpp by coupling it to a fluorescent output. We used mathematical modelling to analyse the intracellular dynamics of ppGpp, its fluorescent reporter, and cell growth in normal and fatty acid-producing *E. coli* lines. The model integrates existing mechanisms of ppGpp regulation and predicts the biosensor response to changes in nutrient state. In particular, the model predicts that excessive stimulation of fatty acid production depletes fatty acid intermediates, downregulates growth and increases the levels of ppGpp-related fluorescence.

**Conclusions:**

Our analysis demonstrates that the ppGpp sensor can be used for early detection of nutrient limitation during cell growth and for testing productivity of engineered lines.

**Electronic supplementary material:**

The online version of this article (10.1186/s12918-017-0490-5) contains supplementary material, which is available to authorized users.

## Background

The efficient production of biofuels, recombinant proteins and other useful compounds in *E. coli* cells requires the optimization of metabolic fluxes and growth conditions [1–3]. The uncontrolled consumption of essential nutrients might cause early cessation of growth and reduction of product yields [1, 2]. Interestingly, cells possess a natural mechanism of sensing of nutrient limitation related to the production of the second messenger guanosine tetraphosphate, **ppGpp** (an “alarmone”). This signalling pathway might be useful for the control of biotechnological processes. ppGpp is an early sensor of nutrient limitation, which directly controls bacterial growth by binding to RNA polymerase bound to ribosomal RNA (rRNA) gene promoters P1 and P2 [4–10]. Binding of ppGpp decreases the life time of short lived open complexes that RNA polymerase forms with P1 and P2 promoters. By inhibiting RNA polymerase activity ppGpp adjusts rRNA biosynthesis to available nutrient levels [4–6, 11, 12]. Regulation of the P1 and P2 promoters by ppGpp is fast (minutes) and covers a wide dynamic range of P1, P2 activities [6, 11]. This suggests the possibility of transmitting changes in ppGpp concentrations into a P1, P2-coupled fluorescent output, which would allow continuous monitoring of transcriptional activity of ppGpp inside the cells. This approach is different from previously developed chemosensors, which were used for end time-point measurements of ppGpp concentration in bacterial lysates [13–15]. The practical application of these chemosensors was limited due to small spectral shift in their fluorescence upon ppGpp binding and the requirement to synthesise these complicated organic compounds [13, 16, 17]. Using the natural mechanism of ppGpp sensing via modulation of P1, P2 activity should overcome the limitations of these previously designed ppGpp chemosensors. Here we use mathematical modelling to analyse the intracellular kinetics of ppGpp and to design a ppGpp-based biosensor that reports ppGpp concentration and thus serves to indicate poor intracellular nutritional status. Next we explore the capacity of the biosensor to respond to dynamic changes in intracellular nutrient state during batch growth of fatty acid-producing and non-producing *E. coli* cells.

**Fig. 1:**
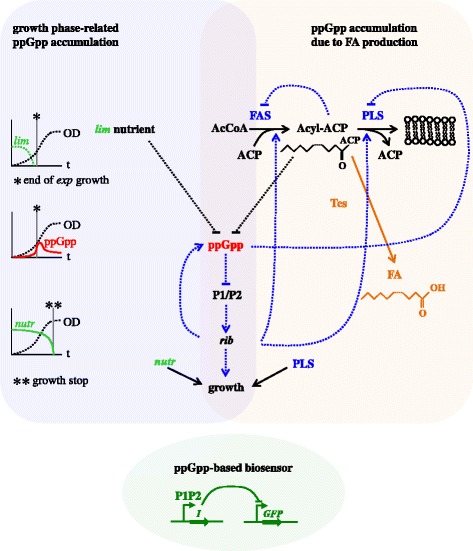
The scheme for ppGpp signalling, FA production and ppGpp sensor included in the model. The **left colour box** illustrates the relationships between ppGpp and growth. ppGpp accumulates at the end of *exp.* growth phase [6, 8], marked by asterisk. This is described in the model through a depletion of *exp.* phase-limiting nutrient *lim* (e.g., main carbohydrate). Increase of ppGpp inhibits the rRNA biosynthesis from P1/P2 promoter [6, 25], which slows down the growth [12]. The termination of growth in stationary (*stat*) phase was described in the model by the depletion of growth-supporting nutrient *nutr* (e.g., secondary carbohydrate) (double asterisk). The decrease of ppGpp concentrations in *stat* phase [6, 8] was described in the model through downregulation of ribosome-mediated synthesis of ppGpp [5]. The **right colour box** illustrates the relationship between ppGpp and fatty acids. In normal cells Acyl-ACP product of the fatty acid synthesis (FAS) is consumed for membrane PL synthesis (PLS). But in FA-producing cells Acyl-ACP is diverted for the synthesis of FA by thioesterase (Tes) enzyme (orange) [22]. Excessive production of FA leads to Acyl-ACP depletion, which stimulates accumulation of ppGpp [10]. In addition to inhibiting growth, ppGpp inhibits PLS flux (through inhibition of the key enzyme PlsB, [26]), causing transient accumulation of Acyl-ACP, which downregulates FAS flux through a feedback inhibition of key FAS enzymes by Acyl-ACP [20, 26]. FAS and PLS fluxes are also inhibited in *stat* phase due to decrease in protein synthesis at low *rib* [22]. The growth is additionally inhibited by PLS decrease [27]. The ppGpp-mediated regulations are shown in blue. The **bottom colour box** shows the proposed ppGpp-based biosensor. It includes the expression of transcriptional inhibitor *I* from the P1/P2 promoter and repression of GFP expression by *I*

Fatty acids (**FA**s) are potential biofuels, which can be synthesised by engineered *E. coli* lines overexpressing a thioesterase (**Tes**) enzyme. Tes hydrolyses the thioester bond in Acyl-ACP molecule [2, 18, 19]. **Acyl-ACP** (long-chain FA linked to activated acyl carrier protein, hereafter simply called ACP) is the primary product of fatty acid synthesis (**FAS**) [10, 20, 21], which is naturally used by cells for phospholipid (**PL**) production (Fig. 1). Additionally to FA synthesis, Acyl-ACP can be diverted for the production of other types of biofuels, such as long chain alkyl esters and alkanes in engineered lines [22]. Alkanes represent the most desirable biofuel, with the highest energy density; however, attempts to engineer alkane-producing organisms have been hampered by low yields and high contamination with fatty alcohols [23, 24]. It was proposed that it might be more practical to use chemical production of alkanes from FA, because of the much higher yields of FA in cells, and economy of cellular resources which would otherwise be required for expression of alkane-synthesising pathways [2]. Therefore the production of FAs in Tes-overexpressing (**Tes-ox**) lines of *E. coli* represents an important technological step in the biosynthesis of alkanes [23]. However, lines with high Tes levels have decreased FA yields and growth, which might be related to depletion of the Acyl-ACP pool required for membrane biosynthesis [2]. It was suggested that the consumption of Acyl-ACP for the production of Acyl-ACP-derived products should be carefully monitored in order to achieve high yields [19, 22]. A natural mechanism of sensing Acyl-ACP depletion is mediated by the accumulation of ppGpp due to decreased activity of the ppGpp hydrolase SpoT [5, 10]. This suggests that a ppGpp biosensor might be used for diagnostics of the productivity of FA-producing lines.

Nutrient depletion increases ppGpp levels through various mechanisms [5]. Thus, amino acid starvation upregulates ppGpp synthase RelA, while carbohydrate and fatty acid starvation downregulates ppGpp hydrolase SpoT [5]. Therefore, nutrient (in particular, carbohydrate) limitation at the end of fast exponential growth phase (hereafter called ***exp***) leads to an increase of ppGpp concentration [6, 8]. This in turn slows down the growth. For example, SpoT mutants with high ppGpp levels have reduced P1, P2 activities and slower growth [12]. Additionally to growth phase-specific accumulation of ppGpp, it is expected to be elevated in high FA-producing lines due to Acyl-ACP depletion [5, 10]. Here we model the changes in the levels of ppGpp and ppGpp-based fluorescence (a biosensor) in normal and FA-producing *E. coli* cells. We explore the potential effects of biosensor parameters on its performance and demonstrate that the sensor might be used for early detection of nutrient limitation during *E. coli* growth.

## Methods

1. Model construction

Here we model changes in the concentrations of ppGpp and its fluorescent reporter GFP (the biosensor) in *E. coli* cells. We consider 2 types of nutrient limitation, which might increase intracellular ppGpp levels: growth phase-specific or non-specific (Fig. 1). The first type is related to a depletion of growth-limiting nutrient (e.g., carbohydrates) in the growth medium at the end of *exp.* growth phase. The second type is related to a depletion of essential molecules inside a cell due to their excessive diversion into the production of biotechnological products (e.g.*,* depletion of Acyl-ACP during fatty acid (**FA**) production; [2], Fig. 1). We use the model to describe and explain the existing data on ppGpp and growth in normal and FA-producing cells ([2, 6]; Results) and to explore the applicability of a ppGpp biosensor for the control of growth conditions and optimization of FA production. The parameters of the model were estimated based on available data as described in Additional file 1. The analysis of parameter sensitivity demonstrated that the model is robust to parameter variation (Additional file 1: Fig. S1). The model consists of 9 ordinary differential equations presented below.

The processes included in the model are summarised on Fig. 1 and described below. Briefly, we model the mutual relationship between ppGpp and growth phases. The increase of ppGpp concentration [6, 8] at the end of *exp.* growth was described by the depletion of *exp.* phase-limiting nutrient *lim* (e.g., main carbohydrate, see Results). The decrease in the growth rate during *exp.* to *stat* phase transition is described through ppGpp-mediated decrease of ribosomal synthesis [6, 25]. The termination of growth in *stat* phase is described by the depletion of a second, growth-supporting nutrient *nutr* (e.g., secondary carbohydrate, which is released into the medium during *exp.* phase; Results).

The model also describes ppGpp accumulation due to a depletion of Acyl-ACP product of FAS during FA production (Fig. 1). The accumulation of ppGpp inhibits membrane phospholipids synthesis (**PLS**), FAS, and growth through several parallel mechanisms in our model (Fig. 1). This includes inhibition of the key PLS enzyme PlsB by ppGpp [26], causing transient accumulation of Acyl-ACP and inhibition of FAS flux by Acyl-ACP [20, 26]; downregulation of FAS and PLS fluxes and growth by low ribosomal activity ***rib*** (relative number of active ribosomes; Fig. 1); and inhibition of growth by decreased PLS flux in high FA-production lines [27].

In addition to ppGpp, FA and growth, the model analyses the kinetics of GFP reporter of ppGpp in FA-producing and non-producing lines. The ppGpp reporter is designed using the natural property of ppGpp to inhibit transcription from P1 and P2 promoters, responsible for rRNA biosynthesis (Fig. 1; [6, 11, 25]). P1 and P2 promoters are regulated in a similar way in *E. coli* cells [6, 11], so we considered a tandem of the ribosomal P1 and P2 promoters as a single entity (hereafter called the **P1/P2** promoter) in our model. To transmit the increase of ppGpp levels to increase of a fluorescent signal, an artificial inhibitor *I* (e.g., tet-repressor TetR or lac repressor LacI) is expressed from the P1/P2 promoter. Expression of GFP protein is from an *I*-repressible promoter (e.g., TetR- or LacI-inhibited promoter; Fig. 1). The use of rapidly degraded variants of *I* and GFP proteins ensures that the biosensor reports dynamic changes in the metabolic state of the cells [28, 29], as we discuss in Results. The levels of intracellular compounds are determined in our model by their synthesis and degradation. Their dilution due to cell division was ignored due to its slow rate (less than 0.01 min^−1^, [8]).

The model equations are presented below.

1. Acyl-ACP and FA production

The intracellular kinetics of the key intermediate of fatty acid metabolism Acyl-ACP is described in our model as:

1 $$ \frac{dAcyl\hbox{-} ACP}{dt}={V}_{FA S}-{V}_{PLS}-{k}_{fa}\cdot {V}_{FA} $$

Where *V*_*FAS*_ and *V*_*PLS*_ are the intracellular rates of FAS and PLS (in μM/min); *V*_*FA*_ is the rate of FA production in a cell culture, normalized to cell number N (in mg/l/min/OD); and *k*_*fa*_ is a volume coefficient for re-calculation of the *V*_*FA*_ rate into intracellular units of μM/min (Additional file 1).

The rate of FAS is described by the simplified lump equation, which includes the Michaelis-Menten dependence of FAS rate on concentration of substrates - AcCoA and ACP protein and feedback inhibition of FAS rate by Acyl-ACP product [26]:

1' $$ {V}_{FAS}={V}_{m- FAS}\cdot rib\cdot \frac{ACP}{ACP+{K}_{m- ACP}}\cdot \frac{AcCoA}{AcCoA+{K}_{m- AcCoA}}\cdot \frac{1}{1+ Acyl\hbox{-} ACP/{K}_{i- Acyl- ACP}} $$

Where AcCoA and ACP are concentrations of acetyl-CoA and free active ACP protein; Acyl-ACP is the total concentration of long-chain acyl product of FAS.

We assumed that FAS and PLS fluxes and growth rate are proportional to the ribosomal activity *rib* due to the dependence of the protein synthesis on *rib*.

PLS rate is assumed to be determined by the rate of the first committed enzyme, PlsB, with Michaelis-Menten dependence on Acyl-ACP substrate and inhibition by ppGpp [19, 26]:

1'' $$ {V}_{PLS}={V}_{m- PLS}\cdot rib\cdot \frac{Acyl\hbox{-} ACP}{Acyl\hbox{-} ACP+{K}_{m- Acyl- ACP}}\cdot \frac{1}{1+{\left( ppGpp/{K}_{i- ppGpp}\right)}^n} $$

The use of the Hill function for the inhibition of PLS by ppGpp is motivated by existing data on multiple levels of negative regulation of PlsB and related enzymes by ppGpp [22].

The rate of FA production is described by Michaelis-Menten kinetics of thioesterase Tes, which hydrolyses thioester bond in the molecule of Acyl-ACP and releases free FA:

$$ {V}_{FA}={V}_{tes}\cdot \frac{Acyl\hbox{-} ACP}{Acyl\hbox{-} ACP+{K}_{m- tes}} $$ (1″‘).

The activity of Tes (*V*_*Tes*_) depends on Tes concentration. In simulated Tes-ox and normal lines *V*_*Tes*_ values were estimated from the measured FA yields ([2]; see Results).

The rate of the total FA production by a cell culture is described as:

2 $$ \frac{dFA}{dt}=N\cdot {V}_{FA} $$

where N is a cell number.

2) Cell growth

Our model describes the kinetics of cell growth (cell number, N) [2, 6], which affects nutrient levels and FA yields. The accumulation of ppGpp at the end of *exp.* phase (Fig. 1) was described by the depletion of a limiting nutrient (variable *lim*, eqs. 5, 6, see below). Increase of ppGpp in turn leads to decrease of the ribosomal activity (eqs. 7, 7′) and inhibition of growth (eq. 3, Fig. 1) during *exp.* to stationary (***stat***) phase transition. The depletion of a second, growth-supporting nutrient (variable *nutr*; eq. 4) determines the cessation of growth and entrance to *stat* phase. Additionally, we assumed that a certain minimal rate of phospholipid synthesis (*V*_*0*_) is required to sustain growth [27]. In addition to limited production of total membrane PL, the membrane composition might be unbalanced in high Tes-ox lines due to higher proportion of unsaturated FA, which was suggested to be a key factor of FA toxicity and growth limitation in high Tes-ox lines [30]. In our model, the growth limitations in high Tes-ox lines [2] were collectively accounted by restricting growth rate at low rates of PLS (*V*_*PLS*_) (eq. 3‘below). The cell growth is described as:

3 $$ \frac{dN}{dt}={v}_g\cdot N $$

3' $$ {v}_g={K}_{gr}\cdot nutr\cdot rib\cdot {V}_{PLS}/\left({V}_{PLS}+{V}_0\right) $$

Where *v*_*g*_ is the growth rate (in min^−1^) and N is a cell number, expressed in units of OD measured at a wavelength of 600 nm.

The kinetics of *nutr* and *lim* depletion was assumed to be proportional to cell number N:

4 $$ \frac{dnutr}{dt}=-{k}_{nutr}\cdot N\cdot \frac{nutr}{nutr+0.001} $$

5 $$ \frac{d\mathit{\lim}}{dt}=-{k}_{lim}\cdot N\cdot \frac{\mathit{\lim}}{\mathit{\lim}+0.001} $$

where *nutr* and *lim* are relative amounts of the growth-supporting and *exp.* phase-limiting nutrients respectively, initially both set to 1. To avoid negative values of *nutr* and *lim*, their depletion is restricted when their concentrations reach levels of 0.001.

3) ppGpp kinetics

The kinetics of ppGpp is determined by the balance between its synthesis and hydrolysis [5]:

6 $$ \frac{dppGpp}{dt}={k}_{+ ppGpp}\cdot rib- ppGpp\cdot \left({k}_{- ppGpp}\cdot \mathit{\lim}+k{0}_{- ppGpp}\right)\cdot \frac{Acyl\hbox{-} ACP}{Acyl\hbox{-} ACP+{K}_{m- Ac\_ pp}} $$

Here *ppGpp*, *rib* and *lim* are the levels of ppGpp, ribosome activity and limiting nutrient *lim*, respectively. Several molecular mechanisms are integrated during ppGpp synthesis and degradation. ppGpp is synthesises on ribosomes [5, 6], therefore in our model we assumed that the synthesis rate of ppGpp is proportional to the number of active ribosomes *rib* [5, 6], with the rate constants *k*_*+ppGpp*_. We next assumed that depletion of *lim* nutrient at the end of the *exp.* phase slows down ppGpp hydrolysis (rate constant *k*_*-ppGpp*_), presumably via the inhibition of ppGpp hydrolase SpoT [31, 32]. This results in the increase of ppGpp concentration at the end of *exp.* phase in our model ([6, 8], Fig. 1). The background hydrolysis of ppGpp in the absence of *lim* is accounted for by the rate constant *k0*_*-ppGpp*_. Finally, ppGpp levels are upregulated in our model by the depletion of Acyl-ACP, due to the inactivation of SpoT hydrolase activity [5, 10, 21, 33].

4) Ribosomal and P1/P2 promoter activities

The equation for the ribosomal activity (relative number of active ribosomes) *rib* is:

7 $$ \frac{drib}{dt}={k}_{+ rib}\cdot P1P2-{k}_{- rib}\cdot rib $$

where *P*1*P*2 and *rib* are the relative activities of the P1/P2 promoter and the ribosomes. The *rib* synthesis is determined by the rate of rRNA transcription from the P1/P2 promoter [6, 11, 34]; therefore, we assume a linear dependence of *rib* synthesis rate on P1/P2 activity.

Based on the existing data we assumed that during cell growth transcription from P1/P2 promoter is regulated by ppGpp inhibition [6, 11, 25]. P1 and P2 show similar responses to changes in nutrient levels, but the tandem of P1 and P2 promoters (P1/P2) shows a stronger response compared to single P1 and P2 promoters [11]. This was accounted for in the model by using a Hill coefficient *m* = 2 in the equation for P1/P2. The data [6, 11] suggest that P1/P2 activity quickly (in minutes) responds to changes in ppGpp concentration. Therefore transcriptional activity of P1/P2 was expressed via ppGpp by the algebraic equation:

7' $$ P1P2=\frac{1}{1+{\left( ppGpp/{k}_{i-P1P2- ppGpp}\right)}^m} $$

5) ppGpp sensor

ppGpp sensing was implemented through the inhibition of GFP expression by the inhibitor *I*, which is expressed from the P1/P2 promoter (Fig. 1). Since the abundance of most proteins changes much more slowly than the abundance of their mRNAs [28], we assumed that the amount of *I* mRNA is simply proportional to the transcriptional activity of P1/P2 promoter, so that the kinetics of protein *I* is described by the following equation:

8 $$ \frac{dI}{dt}={k}_I\cdot \left(P1P2-I\right) $$

where *I* is the relative amount of the inhibitor *I* (changing from 0 to 1) and *k*_*I*_ is a rate constant of protein *I* degradation. The rate constant of protein *I* synthesis was assumed to be equal to *k*_*I*_ to achieve the maximal level of *I* = 1.

The amount of GFP mRNA is determined by the amount of the inhibitor *I*. The equation for the relative amount of fluorescent GFP protein is:

9 $$ \frac{dGFP}{dt}={k}_{GFP}\cdot \left(\frac{1}{1+{\left(I/{Ki}_I\right)}^l}- GFP\right) $$

where *GFP* is the relative amount of GFP fluorescence (changing from 0 to 1) and *k*_*GFP*_ is the rate constant of GFP protein degradation. The rate constant of GFP synthesis was assumed to be equal to *k*_*GFP*_ to achieve the maximal level of *GFP* = 1. A Hill coefficient *l* = 4 accounts for the formation of tetrameric inhibitor complex (e.g., lacR) on a double-stranded DNA [35].

The constant *Ki*_*I*_ for inhibition of GFP expression by the relative amount of the inhibitor *I* integrates two unknown parameters of the system: the absolute expression level of *I* and its inhibition strength. *Ki*_*I*_ was varied as discussed in Results, with the optimal value of *Ki*_*I*_ = 0.1. The system of ODEs was solved using MATLAB, integrated with the stiff solver ode15s (The MathWorks UK, Cambridge). The MATLAB code of the model is provided in Additional file 1.

## Results and discussion

*E. coli* cells are commonly used as cell factories for the production of useful products, such as FA [1, 2, 18]. But the redirection of nutrients from essential metabolic pathways often slows down cell growth and limits product yields [1–3]. To control the growth conditions during biotechnological applications of *E. coli* cells, we propose to use a biosensor, which couples changes in ppGpp concentrations with a fluorescent GFP-based output (Fig. 1). The increase GFP levels would allow detection of nutrient limitation, which could be used for controlling and optimizing growth conditions. To simulate intracellular dynamics of ppGpp during batch cultivation of *E. coli* cells, we built a mathematical model, which integrates literature data on ppGpp regulation. The model describes the interrelationship between ppGpp, growth and FA production, and simulates the kinetics of GFP reporter of ppGpp, as summarised in the legend of Fig. 1 and discussed below. We used the model to describe and explain the existing data on ppGpp and growth in normal and FA-producing lines [2, 6] and analysed applicability of the sensor for monitoring of ppGpp concentration under various conditions.

### ppGpp kinetics in normal (non-FA-producing) *E. coli* cells

**Fig. 2:**
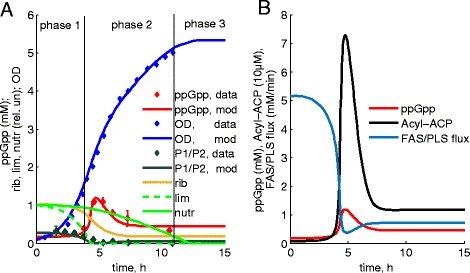
The kinetics of model variables during batch growth of normal, non-FA producing lines of *E. coli* cells. **a**. The blue line and symbols show a growth curve (OD units) in the model and published data [6]. Three phases of growth are indicated by black vertical lines: 1-*exp.* phase, 2-*exp.*-to-*stat* transition phase, 3-*stat* phase. ppGpp concentrations in the model and published data [6] are shown by the red line and symbols respectively. The end of phase 1 coincides with an increase of ppGpp concentration and the depletion of limiting nutrient *lim* (green dotted line). The end of phase 2 coincides with the depletion of the growth-supporting nutrient *nutr* (green solid line). The transcriptional activity of P1/P2 promoter (normalized to maximum) is shown by black solid line and black symbols for the model and the data [6] respectively. The relative ribosomes number is shown by the orange line. The data on ppGpp kinetics were quantified based on available measurements of ppGpp concentrations [8]. **b**. The kinetics of Acyl-ACP, ppGpp and FAS/PLS flux are shown by black, red and blue colours respectively

Cell growth in batch cultures is widely used in biotechnology, and growth can be described by three main phases [36, 37]. The first phase, *exp.* growth, is characterized by an exponential increase of the cell number (Fig. 2a). During the second phase, growth is gradually slowing down, while cells move from *exp.* to *stat* phase (Fig. 2a). The third, *stat* phase is characterized by the absence of growth (Fig. 2a). The existing data suggest that the end of *exp.* phase coincides with increase of ppGpp concentration ([6, 8], Fig. 2a). Therefore, the slowing down of growth in phase 2 appears to be a consequence of ppGpp increase, presumably related to nutrient depletion during *exp.* phase. However, the cell number keeps increasing during the second phase (Fig. 2a), and both the first and second phases are characterized by high accumulation of biotechnological products, such as FA in engineered lines [2, 3, 18]. This suggests that despite a depletion of an *exp.* phase-limiting nutrient (called *lim* in our model), the medium has sufficient amount of other nutrients to sustain growth. To describe the observed kinetics of growth and ppGpp, we implemented a two-factor depletion scheme in our model (Fig. 1). We assumed that sequential depletion of two nutrient factors *lim* and *nutr* determines the growth phases. The observed increase of ppGpp concentration at the end of phase 1 was explained in our model by the inhibition of ppGpp hydrolysis upon depletion of *lim* [31, 32] (Fig. 2a). The *lim* factor might be represented by a carbohydrate nutrient (e.g. glycerol for [2, 6] conditions) or some other components of the medium, whose depletion limits *exp.* growth. The decline of growth rate during phase 2 is explained in our model through ppGpp-mediated inhibition of rRNA transcription from P1/P2 promoter (Fig. 1), in agreement with the P1/P2 data (Fig. 2a; [6]). The termination of phase 2 is described in our model through the depletion of a second nutrient *nutr*, causing the cessation of growth and entrance to *stat* phase (Fig. 2a). *nutr* might represent a secondary carbohydrate source, such as acetate, which is released into the medium during phase 1. Alternatively, depletion of other nutrients might limit cellular growth during the two phases. The nature of the limiting factor might vary between different experiments [1, 36, 37]. However our two-factor phenomenology provides a good fit to growth data [2] (see below). Notably, after reaching its maximum, ppGpp concentration decreases during phase 2 (Fig. 2a; [6, 8]). The model explains this by a decrease in ribosome-mediated synthesis of ppGpp during phase 2 (Figs. 1 and 2a; [6, 38–40].

The model also predicts the kinetics of Acyl-ACP accumulation during cell growth. Acyl-ACP is produced by FAS and consumed for PL and FA synthesis (Fig. 1). In normal lines the production of free FA is negligible [18]; therefore FAS and PLS fluxes are equal. In our model, FAS and PLS fluxes are downregulated by ppGpp at the beginning of phase 2. Thus, PLS is inhibited by ppGpp directly, which leads to transient accumulation of Acyl-ACP and resulting inhibition of FAS by Acyl-ACP (Figs. 1 and 2b; [19, 20, 26, 41]). In addition, FAS and PLS fluxes are downregulated by low ribosome activity *rib*, which affects protein synthesis and thus metabolic fluxes in our model (Fig. 1). Therefore, the model predicts a transient accumulation of Acyl-ACP due to the inhibition of PLS by ppGpp at the beginning of phase 2, followed by a decrease of Acyl-ACP levels due to a drop of the FAS rate (Fig. 2b).

### Sensing of ppGpp levels in normal *E. coli* cells. Response time of GFP fluorescent output

The synthesis of ppGpp, followed by the fast (~minutes) ppGpp-mediated inhibition of the transcription from P1/P2 promoter provides an early sensing of nutrient limitation in bacterial cells (Fig. 1; [6, 11]). Based on this natural mechanism, in our biosensor we express the inhibitor *I* (e.g., TetR or LacI) of GFP expression under the control of the P1/P2 promoter (Fig. 1). An increase of ppGpp downregulates the transcription from P1/P2, leading to a decrease of *I* levels and expression of GFP protein. The response of the sensor to dynamic changes in ppGpp concentrations can be accelerated by using rapidly degraded versions of *I* and GFP proteins [28, 29], as discussed below.

**Fig. 3:**
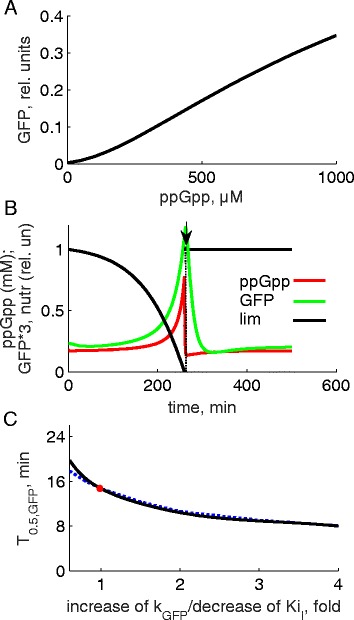
Modelling of the ppGpp-based biosensor in normal, non-FA-producing *E. coli* lines. **a**. Dependence of steady state GFP fluorescence on ppGpp concentrations. Each data point was calculated at different ppGpp concentration. **b**. Timecourses of ppGpp, GFP and *lim* in the model during nutrient upshift (shown by arrow) at the end of phase 1. **c**. Dependence of the response time of GFP fluorescence (*T*_*0.5,GFP*_) on the fold increase of the rate constant *k*_*GFP*_ of GFP protein degradation (black) or fold decrease of the rate constant *Ki*_*I*_ of the inhibition of GFP expression by the inhibitor *I* (blue). *T*_*0.5,GFP*_ was calculated as the time required for 2-fold decrease of GFP fluorescence after the nutrient upshift. Red circle indicates the value of *T*_*0.5,GFP*_ corresponding to *k*_*GFP*_ = 0.1 min^−1^ and *Ki*_*I*_ = 0.3 used in **a, b**

The model demonstrates that the double negative regulation of GFP expression by ppGpp (Fig. 1) results in a strong correlation between steady state levels of ppGpp and GFP (Fig. 3a). This suggests that GFP can be used as a reporter of ppGpp concentration over a wide range of observable ppGpp concentrations (0.05–1 mM; [6, 8]). Since ppGpp inhibits growth, GFP fluorescence indicates the degree of growth inhibition. The ppGpp sensor might be used for monitoring of intracellular metabolic state in various biotechnological applications. In the next section we applied it for diagnostics of FA-producing lines. The sensor can also be used for exploring the nature of the growth-limiting nutrient *lim*. Thus, the model predicts that if a nutrient limits growth, its restoration at the end of phase 1 would result in a quick decrease of ppGpp-related GFP fluorescence (Fig. 3b). The response time of GFP fluorescence, T_0.5,GFP_ (half-life of GFP after nutrient upshift) depends on the model parameters. The model predicts that accelerated degradation of GFP protein can substantially shorten the GFP response time (Fig. 3c). Therefore, the use of rapidly degraded fluorescent reporters is desirable. Similarly, an increase of the inhibition strength of GFP expression by inhibitor *I* can also shorten the T_0.5,GFP_ (Fig. 3c). Therefore manipulation of the expression level of *I* seems to be the most practical approach for optimizing biosensor performance, as further discussed below.

### ppGpp kinetics in FA-producing lines of *E. coli*

**Fig. 4:**
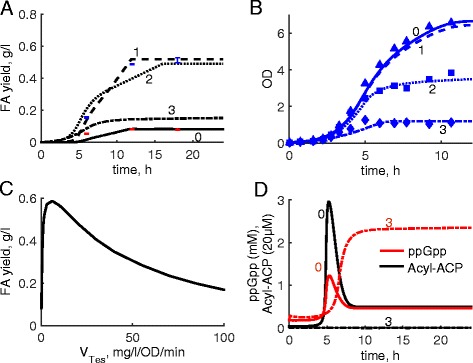
Modelled kinetics of FA production during batch growth of *E. coli* cells. Kinetics of FA yields (**a**, in mg FA per 1 l of cell culture) and growth (**b**, in OD units) in cell cultures, expressing different amounts of thioesterase Tes. **a, b**. Solid, dashed, dotted and dashed-dotted lines correspond to the simulated control line 0 and lines 1, 2, 3 (indicated by numbers) with Tes activity of 0.08, 1.6, 19 and 110 mg/l/OD/min respectively. Data points are redrawn from [2] and correspond to lines 0 (red) and 1 (blue) (**a**) and lines 0, 2, 3 (**b**). **c**. Dependence of modelled FA yield of cell cultures after 24 h of growth on the levels of Tes activity. **d**. Intracellular kinetics of ppGpp (red) and Acyl-ACP (black) in the control line 0 and high Tes line 3, shown by solid and dashed-dotted lines respectively

We next used the model to analyse the efficiency of FA production in Tes-ox lines of *E. coli* with different expression levels of Tes [2]. Tes-ox lines were simulated by fitting Tes activities (*V*_*Tes*_) to the measured FA yields of growing cell cultures (Fig. 4a; [2]). Figure 4a,b shows simulated kinetics of FA accumulation and cell growth in the control line 0 (only endogenous Tes is present) and Tes-ox lines 1, 2, 3 with low, medium and high levels of over-expressed Tes. The model predicts that increased expression of Tes should initially increase FA yields, but only until a certain critical level of Tes, above which a decrease of FA yields is observed (Fig. 4a,c). For example, high-Tes line 3 has 3-fold lower FA yield compared to line 1 (Fig. 4a), which corresponds to experimental observations on lower FA yields in high Tes lines [2]. The model explains the reduction of FA yields in high Tes lines by depletion of Acyl-ACP levels, which is predicted to increase the accumulation of ppGpp (Figs. 1 and 4d). High ppGpp in turn should reduce growth rates, in agreement with the data (Fig. 4b). Additionally, a strong pull of Acyl-ACP towards FA production reduces synthesis of membrane lipids, which further downregulates growth in high Tes lines in our model (Fig. 1).

### Using the ppGpp-based biosensor for diagnostics of the efficiency of FA-producing lines

**Fig. 5:**
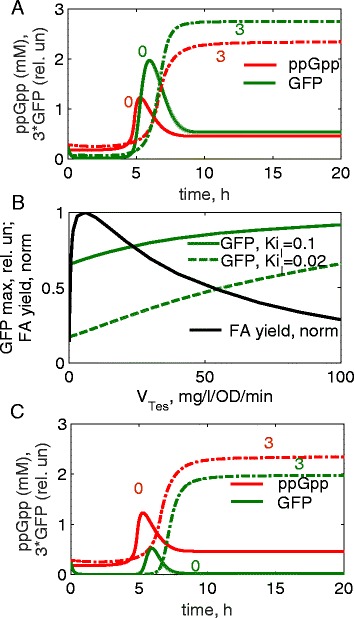
Using the ppGpp sensor for diagnostics of low efficiency of FA-producing lines. **a**. Simulated GFP fluorescence (green) follows ppGpp kinetics (red) during batch cultivation of *E. coli* cells; *Ki*_*I*_ = 0.1. Solid and dashed-dotted lines correspond to the control line 0 and high Tes line 3 (indicated by numbers) with Tes activity of 0.08 and 100 mg/l/OD/min respectively. **b**. Dependence of the maximal levels of GFP fluorescence during batch cultivation on the Tes activity. Green solid and dashed green lines correspond to *Ki*_*I*_ = 0.1 and *Ki*_*I*_ = 0.02 respectively. The black line shows FA yields after 24 h of cell growth, normalized to maximum. **c**. Kinetics of GFP fluorescence (green) and ppGpp concentrations (red) under increased strength of GFP inhibition (*Ki*_*I*_ = 0.02). Line designations are the same as in **a**

The model predicts that depletion of Acyl-ACP in high-Tes lines should lead to increased ppGpp levels (Figs. 1 and 4d), which downregulates growth and FA yields. In addition, GFP levels are predicted to increase in parallel to ppGpp in control and Tes-ox lines (Fig. 5a). This suggests that elevated GFP levels might serve as an indicator of low efficiency of FA production. Indeed, the peak levels of GFP inversely correlate with FA yields at high Tes levels (Fig. 5b). Therefore laborious methods of FA quantification in testing the efficiency of Tes-ox lines might be replaced by measurements of GFP fluorescence. The model analysis further suggests that the parameters of the ppGpp sensor affect the steepness of the dependence of GFP fluorescence on Tes levels. In particular, strengthening of the inhibition of GFP expression by *I* (e.g., by increasing of *I* expression) increases the steepness (Fig. 5b). Therefore, the increased expression of *I* improves the sensitivity of the sensor to the variations in Tes levels, in parallel to the improvement of GFP response time (Figs. 3c and 5b). However, increased inhibition of GFP expression by *I* also leads to a reduction of the total level of GFP fluorescence (Fig. 5c). Therefore there is a trade-off between the rate and the amplitude of the GFP response. This should be kept in mind during the optimisation of the sensor responsiveness, so that the total GFP signal might be rather low but within the detection limit. The level of GFP fluorescence might be further increased by using specially designed bright mutants of GFP with 20–35-fold higher intensity compared to standard GFP [42].

In addition to testing FA productivity, the biosensor might be used for optimization of FA production. To achieve this, a specific version of the sensor might be developed, which includes a feedback downregulating Tes expression at high ppGpp levels, potentially by using ppGpp-sensitive transcription factors.

Existing methods of genetic manipulations should be sufficient to perform the proposed engineering of the biosensor. In particular, recombination with serine integrases might be especially useful, because it allows insertion and removal of DNA fragments, if required [43]. This property of serine integrase is related to their ability to reverse the directionality in presence of recombination directionality factors (RDFs). Therefore, combinations of specific genes, promoters and ribosome-binding sites might be tested to optimize biosensor performance.

## Conclusions

Based on existing data we built a mathematical model of intracellular kinetics of ppGpp during batch growth of *E. coli* cells. To monitor nutrient limitation, we designed a ppGpp biosensor, which couples changes in ppGpp concentrations and P1/P2-mediated transcription to a fluorescent output. The model demonstrates that the biosensor can sense a wide range of intracellular ppGpp concentrations and dynamically respond to perturbations of bacterial metabolism, such as nutrient upshifts and *log* to *stat* phase transition. The model predicts that both types of nutrient limitations, either related to nutrient depletion due to the increase of cell number (growth phase-specific) or to high consumption of Acyl-ACP for synthesis of FA in engineered lines (non-growth phase-specific) can be easily sensed by the ppGpp sensor. The use of quickly-degraded variants of GFP protein would increase the responsiveness of ppGpp sensing, which can be additionally adjusted by changing the expression level of the inhibitor of GFP expression. We further demonstrate that the level of ppGpp-dependent fluorescence inversely correlates with FA yields, suggesting that the sensor might be a useful instrument in testing FA productivity of engineered lines of *E. coli*.

## Additional file

Additional file 1: Figure S1. Model equations with estimation of the model parameters. Table with parameter values. Analysis of the parameter sensitivity. Model code in Matlab. (DOC 348 kb)

## Abbreviations

ACP: Activated acyl carrier protein
Acyl-ACP: Acyl-FA bound to ACP
*exp.* phase: exponential growth phase
FA: Fatty acid
FAS: Fatty acid synthesis
GFP: Green fluorescent protein
OD: Optical density
P1/P2: ribosomal RNA gene promoter
PL: Phospholipids
PLS: Phospholipids synthesis
PlsB: glycerol-3-phosphate acyltransferase
ppGpp: guanosine tetraphosphate
RelA: ppGpp synthase
*rib*: ribosomal activity (relative number of active ribosomes)
rRNA: ribosomal RNA
SpoT: ppGpp hydrolase
*stat* phase: stationary growth phase
Tes: Thioesterase
Tes-ox: Tes-overexpressing line
