# Supplementary material for: Monitoring of nutrient limitation in growing E. coli: a mathematical model of a ppGpp-based biosensor
Source: BMC Syst Biol. 2017 Nov 21;11:106. doi: 10.1186/s12918-017-0490-5 (PMC5697348; doi:10.1186/s12918-017-0490-5)
Supplement: Additional file 1: Figure S1. — Model equations with estimation of the model parameters. Table with parameter values. Analysis of the parameter sensitivity. Model code in Matlab. (DOC 348 kb) [file 12918_2017_490_MOESM1_ESM.doc]

**Monitoring of nutrient limitation in growing *E. coli*: A mathematical model of a ppGpp-based biosensor.**

*Alexandra Pokhilko*

***Table of contents***

Model equations with estimation of the model parameters p.1

Table S1. Parameter values p.5

Analysis of the parameter sensitivity p.5

Figure S1 p.6

References p.6

Matlab code of the mode p.7

***Model equations with estimation of the model parameters***

1. Acyl-ACP and FA production

Acyl-ACP production rate is:

$\frac{dAcyl\text{-}ACP}{dt}=V_{FAS}-V_{PLS}-k_{fa}\cdot V_{FA}$ (1),

A volume coefficient *k_fa_* for re-calculation of the *V_FA_* rate of FA production in a cell culture (in mg/l/min/OD) into intracellular units of µM/min was estimated as 4400 µmol∙OD/mg, assuming that 1 unit of optical density (OD) corresponds to 10^9^ cells/ml, cell volume is 10^-15^ l and the molecular weight of an average FA (C14) is 228 g/mol (<http://kirschner.med.harvard.edu/files/bionumbers/fundamentalBioNumbersHandout.pdf>).

FAS rate is:

$V_{FAS}=V_{m\_FAS}\cdot rib\cdot\frac{ACP}{ACP+K_{m\_ACP}}\cdot\frac{AcCoA}{AcCoA+K_{m\_AcCoA}}\cdot\frac{1}{1+Acyl\text{-}ACP/K_{i\_Acyl-ACP}}$ (1')

The Michaelis-Menten constants for the dependence of FAS rate on AcCoA and ACP were estimated from the data on the key enzymes ACC, fabH and malonyl-ACP transacylase (fabD), incorporating AcCoA and ACP into fatty acid intermediates : *Km_AcCoA* = 40 µM and *Km_ACP* = 5400 µM . The inhibition constant for Acyl-ACP is *Ki_Acyl-ACP* = 50 µM . The cellular AcCoA levels are substantially higher than the value of *Km_AcCoA*, and AcCoA becomes depleted only at the end of stationary phase . Therefore in our model we simply fixed AcCoA content to its mid-*log* level of 325 µM . The amount of free ACP was determined from the conservation of the total concentration of active ACP (*ACP_tot_*) as

*ACP_tot_ = ACP+Acyl-ACP*, where *ACP_tot_* =114 µM .

PLS rate is:

$V_{PLS}=V_{m\_PLS}\cdot rib\cdot\frac{Acyl\text{-}ACP}{Acyl\text{-}ACP+K_{m\_Acyl-ACP}}\cdot\frac{1}{1+(ppGpp/K_{i\_ppGpp})^{n}}$ (1'')

The Michaelis-Menten constant of PlsB for Acyl-ACP is *Km_Acyl-ACP* = 70 µM . The Hill coefficient *n* and the inhibition constant *Ki_ppGpp* were fitted to data on a sharp increase of Acyl-ACP (from ~1 to 60 µM) upon 4-fold increase of ppGpp levels in ppGpp-overexpressing lines , giving *n*=3 and *Ki_ppGpp* = 45 µM.

The maximal rate of FAS (*Vm_FAS*) during *log* growth of normal lines of *E. coli* was estimated assuming that 9% of cellular dry weight is fat and cell mass is 1 g per 1 ml volume . This gave FAS flux of 0.09 g of fat/ml/*t_d_*, where *t_d_* is a doubling time. Using a molecular weight of 228 g/mol for the FA product of FAS and *t_d_* ~ 80 min for a glycerol-based medium , the flux can be estimated as ~5 mM/min. The values of *Vm_FAS* and *Vm_PLS* were fitted to get the above value of steady state FAS flux and an estimated level of Acyl-ACP ~1 µM during the *log* phase (~ 0.1 % of the *ACP_tot_*, ), which gave *Vm_FAS* = 0.28 M/min and *Vm_PLS* = 28 M/min.

FA production rate in a cell (in mg/l/min/OD) is:

$V_{FA}=V_{tes}\cdot\frac{Acyl\text{-}ACP}{Acyl\text{-}ACP+K_{m\_tes}}$ (1''')

The thioesterase activity *V_tes_* was determined from the FA production data (Fig. 4, Results).

The rate of FA production by the whole culture is:

$\frac{dFA}{dt}=N\cdot V_{FA}$ (2)

The Michaelis-Menten constant for thioesterase Tes is *Km_Tes*= 16.6 µM . All Tes-ox lines in including the control were in a fadD null background to ensure that synthesized FAs are not degraded via β-oxidation pathways. Therefore, the control line produced noticeable amounts of FA in (see Results), in contrast to wild-type strains of *E. coli*, where FA flux is normally negligible . In simulations of the wild-type strain we assumed *V_Tes_*=0.

1. Cell growth

The kinetics of cell number N (in OD units) is described as:

$\frac{dN}{dt}=v_{g}\cdot N$ (3)

$v_{g}=K_{gr}\cdot nutr\cdot rib\cdot V_{PLS}/(V_{PLS}+V_{0})$ (3')

The value of V_0_=2 µM/min was fitted to OD kinetics in Tes-ox lines . The growth rate constant *K_gr_* and initial cell number (*N(0)*) were fitted to OD kinetics: *K_gr_* = 0.0125 min^-1^ and *N(0)* = 0.1 for and *K_gr_* = 0.015 min^-1^ and *N(0)* = 0.04 for .

The kinetics of growth-supporting and *log* phase-limiting nutrients *nutr* and *lim* is:

$\frac{dnutr}{dt}=-k_{nutr}\cdot N\cdot\frac{nutr}{nutr+0.001}$ (4)

$\frac{dlim}{dt}=-k_{lim}\cdot N\cdot\frac{lim}{lim+0.001}$ (5),

The values of parameters *k_nutr_* and *k_lim_* were fitted to OD and ppGpp data respectively , giving *k_nutr_* = 0.00042 min^-1^ and *k_lim_* = 0.005 min^-1^.

1. ppGpp kinetics

The kinetics of ppGpp is determined by the balance between its synthesis and hydrolysis :

$\frac{dppGpp}{dt}=k_{+ppGpp}\cdot rib-ppGpp\cdot(k_{-ppGpp}\cdot lim+k0_{-ppGpp})\cdot\frac{Acyl\text{-}ACP}{Acyl\text{-}ACP+K_{m\_Ac\_pp}}$ (6)

The dependence of the ppGpp synthesis rate on GDP substrate was ignored, because GDP levels are very stable in *E. coli* cells . Also we assumed that in the absence of amino acid starvation the rate of ppGpp synthesis depends only on ribosome activity *rib*. The parameters of ppGpp synthesis and degradation were fitted to ppGpp and OD kinetic data (; Results), with optimal values *k_+ppGpp_* = 140 µM·min^-1^, *k_-ppGpp_* = 1 min^-1^, *k0_-ppGpp_* = 0.06 min^-1^ , *Km_Ac_pp* = 0.2 µM.

1. Ribosomal and P1/P2 promoter activities

$\frac{drib}{dt}=k_{+rib}\cdot P1P2-k_{-rib}\cdot rib$ (7),

The value of the rate constant *k_+rib_* = 0.036 min^-1^ was chosen to get a maximal *rib* value equal to 1 in simulations of data. The changes in ribosomal activity in our model are rather slow (Results) due to slow degradation of ribosomal RNA, with estimated constant *k_-rib_* of 0.01 min^-1^ .

$P1P2=\frac{1}{1+(ppGpp/k_{i\_P1P2\_ppGpp})^{m}}$ (7')

A value of the inhibition constant *ki_P1P2_ppGpp* of 110 µM was chosen to describe the observed 10-fold decrease of P1/P2 activity upon a ~ 4-fold increase in ppGpp over the basal level of 260 µM during *log* to *stat* transition . The maximal P1/P2 activity in absence of ppGpp is set to 1.

1. ppGpp sensor

$\frac{dI}{dt}=k_{I}\cdot(P1P2-I)$ (8),

$\frac{dP_{GFP}}{dt}=k_{GFP}\cdot(\frac{1}{1+(I/Ki_{I})^{l}}-P_{GFP})$ (9),

We assumed that degradation of the inhibitor *I* is accelerated by using a special tag and estimated *k_I_* based on a half-life of tagged inhibitor ~10 min , which gives a *k_I_* value of ln2/10 min= 0.07 min^-1^.

To ensure fast response of GFP fluorescence to dynamic changes in ppGpp concentrations, rapidly degraded versions of GFP protein can be used, with a half-life of ~7 min . Thus the *k_GFP_* value is ln2/7 min= 0.1 min^-1^. The constant *Ki_I_* for inhibition of GFP expression by the relative amount of the inhibitor *I* was varied as discussed in Results, with the optimal value of *Ki_I_* = 0.1.

Table S1. Parameter values

| parameter | *k_fa_* | *Km_AcCoA* | *Km_ACP* | *Ki_Acyl-ACP* | AcCoA | *ACP_tot_* |
| --- | --- | --- | --- | --- | --- | --- |
| value | 4400 µmol∙OD/mg | 40 µM | 5400 µM | 50 µM | 325 µM | 114 µM |
| ref |  |  |  |  |  |  |
| parameter | *Km_Acyl-ACP* | *Ki_ppGpp* | *n* | *Vm_FAS* | *Vm_PLS* | *Km_Tes* |
| value | 70 µM | 45 µM | 3 | 0.28 M/min | 28 M/min | 16.6 µM |
| ref |  |  |  |  |  |  |
| parameter | V_0_ | *K_gr_* | *k_nutr_* | *k_lim_* | *k_+ppGpp_* | *k_-ppGpp_* |
| value | 2 µM/min | 0.0125 min^-1^ | 0.00042 min^-1^ | 0.005 min^-1^ | 140 µM·min^-1^ | 1 min^-1^ |
| ref |  |  |  |  |  |  |
| parameter | *k0_-ppGpp_* | *Km_Ac_pp* | *k_+rib_* | *k_-rib_* | *ki_P1P2_ppGpp* | *k_I_* |
| value | 0.06 min^-1^ | 0.2 µM | 0.036 min^-1^ | 0.01 min^-1^ | 110 µM | 0.07 min^-1^ |
| ref |  |  |  |  |  |  |
| parameter | *k_GFP_* | *Ki_I_* | *m* | *l* |  |  |
| value | 0.1 min^-1^ | *0.1* | 2 | 4 |  |  |
| ref |  | *varied* |  |  |  |  |

***Analysis of the parameter sensitivity***

To explore the sensitivity of the model to parameter variations we compared the model simulations with the available data on the concentration of ppGpp, P1P2 activity and cell growth (OD). Fig. S1 A demonstrates that 20% increase or decrease of each parameter results in less than 20 % differences in ppGpp amplitude and peak time from the experimentally observed. OD and P1P2 kinetics are similarly close to the experimental observations (Fig. SB-D). The most sensitive parameters are *K_gr_*, *n* and *ki_P1P2_ppGpp*. We conclude that the model is robust to the parameter perturbations.

| A | B |
| --- | --- |
| C | D |

Figure S1. Analysis of parameter sensitivity. The kinetics of ppGpp (A), P1P1 activity (B), OD (C,D) under 20% increase and decrease of each parameter from Table S1 for simulations of (A-C) and (D) data on wild-type and FA-producing lines respectively. The data are the same as on Fig.2A and Fig.4B. The kinetics with optimal (unchanged) parameters is shown by coloured lines. Data points are redrawn from and . The kinetics of OD on D corresponds to high Tes-ox line 3.

***References***

1. Soriano A, Radice AD, Herbitter AH, Langsdorf EF, Stafford JM, Chan S, Wang S, Liu YH, Black TA: **Escherichia coli acetyl-coenzyme A carboxylase: characterization and development of a high-throughput assay**. *Anal Biochem* 2006, **349**(2):268-276.

2. Heath RJ, Rock CO: **Inhibition of beta-ketoacyl-acyl carrier protein synthase III (FabH) by acyl-acyl carrier protein in Escherichia coli**. *J Biol Chem* 1996, **271**(18):10996-11000.

3. Joshi VC, Wakil SJ: **Studies on the mechanism of fatty acid synthesis. XXVI. Purification and properties of malonyl-coenzyme A--acyl carrier protein transacylase of Escherichia coli**. *Arch Biochem Biophys* 1971, **143**(2):493-505.

4. Janssen HJ, Steinbuchel A: **Fatty acid synthesis in Escherichia coli and its applications towards the production of fatty acid based biofuels**. *Biotechnol Biofuels* 2014, **7**(1):7.

5. Takamura Y, Nomura G: **Changes in the intracellular concentration of acetyl-CoA and malonyl-CoA in relation to the carbon and energy metabolism of Escherichia coli K12**. *J Gen Microbiol* 1988, **134**(8):2249-2253.

6. Ray TK, Cronan JE, Jr.: **Acylation of sn-glycerol 3-phosphate in Escherichia coli. Study of reaction with native palmitoyl-acyl carrier protein**. *J Biol Chem* 1975, **250**(21):8422-8427.

7. Heath RJ, Jackowski S, Rock CO: **Guanosine tetraphosphate inhibition of fatty acid and phospholipid synthesis in Escherichia coli is relieved by overexpression of glycerol-3-phosphate acyltransferase (plsB)**. *J Biol Chem* 1994, **269**(42):26584-26590.

8. C. F, C. N, Edwin UH: **Chemical Composition of Escherichia coli**. In: *Escherichia coli and salmonella: Cellular and Molecular Biology.* Edited by F.C. N, vol. 1. Washington, D.C.: ASM Press; 1996: 2.

9. Murray HD, Schneider DA, Gourse RL: **Control of rRNA expression by small molecules is dynamic and nonredundant**. *Mol Cell* 2003, **12**(1):125-134.

10. Buckstein MH, He J, Rubin H: **Characterization of nucleotide pools as a function of physiological state in Escherichia coli**. *J Bacteriol* 2008, **190**(2):718-726.

11. Pollard MR, Anderson L, Fan C, Hawkins DJ, Davies HM: **A specific acyl-ACP thioesterase implicated in medium-chain fatty acid production in immature cotyledons of Umbellularia californica**. *Arch Biochem Biophys* 1991, **284**(2):306-312.

12. Lennen RM, Braden DJ, West RA, Dumesic JA, Pfleger BF: **A process for microbial hydrocarbon synthesis: Overproduction of fatty acids in Escherichia coli and catalytic conversion to alkanes**. *Biotechnol Bioeng* 2010, **106**(2):193-202.

13. Lu X, Vora H, Khosla C: **Overproduction of free fatty acids in E. coli: implications for biodiesel production**. *Metab Eng* 2008, **10**(6):333-339.

14. Potrykus K, Cashel M: **(p)ppGpp: still magical?** *Annu Rev Microbiol* 2008, **62**:35-51.

15. Kaplan R, Apirion D: **The fate of ribosomes in Escherichia coli cells starved for a carbon source**. *J Biol Chem* 1975, **250**(5):1854-1863.

16. Elowitz MB, Leibler S: **A synthetic oscillatory network of transcriptional regulators**. *Nature* 2000, **403**(6767):335-338.

17. Houser JR, Ford E, Chatterjea SM, Maleri S, Elston TC, Errede B: **An improved short-lived fluorescent protein transcriptional reporter for Saccharomyces cerevisiae**. *Yeast* 2012, **29**(12):519-530.

18. Kubitschek HE, Friske JA: **Determination of bacterial cell volume with the Coulter Counter**. *J Bacteriol* 1986, **168**(3):1466-1467.

19. Sezonov G, Joseleau-Petit D, D'Ari R: **Escherichia coli physiology in Luria-Bertani broth**. *J Bacteriol* 2007, **189**(23):8746-8749.

20. Murray HD, Gourse RL: **Unique roles of the rrn P2 rRNA promoters in Escherichia coli**. *Mol Microbiol* 2004, **52**(5):1375-1387.

21. Swint-Kruse L, Matthews KS: **Allostery in the LacI/GalR family: variations on a theme**. *Curr Opin Microbiol* 2009, **12**(2):129-137.

***Matlab code of the model***

ACPtot=114; Km_AcCoA=40; Km_ACP=5400; Km_acylACP=70; AcCoA=325;

Ki_acylACP=50; ki_P1P2_ppGpp=110; Ki_ppGpp=45; kfa=4400; Km_tes=16.6;

kprib=0.036; kmrib=0.01; kI=0.07; kGFP=0.1;

kp_ppGpp=140; km_ppGpp=1; k0m_ppGpp=0.06; Km_Ac_pp=0.2;

V0=2; Vtes=0.08; %Vtes=1.6; %Vtes=19; %Vtes=110;

FASm=280000; VmPLS=28000000; Kgr=0.015; klim=0.005; knutr=0.00042; % Lennen N0=0.04

%Kgr=0.0125; Vtes=0; % Murray N0=0.1

Ki_I=0.1; %Ki_I=0.02;

% units: concentration - mkM, time - min

% y(1) Acyl-ACP

% y(2) I

% y(3) ppGpp

% y(4) rib

% y(5) GFP

% y(6) nutr

% y(7) FA

% y(8) N

% y(9) lim

t_Mur=[183 227 275 318 365 435 637];

ppGpp_Mur=[260.04 401.88 1087.44 1087.44 803.76 661.92 330.96];

pp_er=[78 26 52 104 78 200 52];

t_Mur_prom=[245 288 333 378 423 469 514 588 799]-150;

prom_Mur=[0.63 0.61 0.53 0.37 0.21 0.06 0.11 0.04 0.008]/2;

prom_er=[0.28 0.13 0.12 0.04 0.015 0.037 0.06 0.016 0.005]/2;

t_OD600_M=[191.55 216.83 247.94 300.37 319.99 345.09 370.2 391.58 418.46 441.5 473.97 504.56 548.35 589.97 631.59 680.81 724.25 767.76 801.77]-150;

OD600_M=[0.16 0.22 0.31 0.5 0.7 1 1.29 1.7 2.02 2.39 2.82 3.32 3.8 4. 4.29 4.59 4.69 4.9 5]; % Murray

t_OD_L=[0 0.7 1.6 2.19 2.74 3.3 4.03 5 5.94 6.88 7.73 9.22 10.62]; % Lennen 10

OD_35=[0.04 0.08 0.14 0.25 0.43 0.94 1.76 3.26 4.27 5.18 5.54 6.37 6.59]; %line 0

OD_18=[0.03 0.07 0.13 0.22 0.46 0.85 1.64 2.45 2.84 3.12 2.97 3.46 3.82]; %line 1

OD_34=[0.04 0.08 0.13 0.2 0.32 0.61 0.79 1.25 1.1 1.07 1.14 1.02 1.11];

%line 2

OD_33=[0.03 0.07 0.13 0.21 0.42 0.655 1.536 2.89 3.86 4.41 4.657 5.36 5.96]; %line 3

t_FA=[6 12 18];

FA_wt=[53.4 81.7 78.9]; %line 0

FA=[154.7 487.6 512.4]; % line 1

y0=[0.827941518491469,0.268328682663486,180.201019329569,1.06858327151547,0.0791430674040538,1,0,0.04,1]; %Lennen

%y0=[0.847018571188098,0.268576945995788,181.422911569455,1.01701109409580,0.0776943884199793,1,0,0.1,1]; % Murray

options = odeset('NonNegative',[6,9]);

Tm=24*60;

t=[0 Tm];

[T, Y] = ode15s(@Model_biosens_f,t,y0,options,ACPtot,Km_AcCoA,Km_ACP,Km_acylACP,FASm,VmPLS,AcCoA,kp_ppGpp,km_ppGpp,k0m_ppGpp,Ki_ppGpp,Ki_acylACP,knutr,ki_P1P2_ppGpp,kprib,kmrib,kI,kGFP,Ki_I,Km_tes,Vtes,Kgr,klim,Km_Ac_pp,V0,kfa);

acp=ACPtot-Y(:,1);

PL_rate=VmPLS*Y(:,4).*Y(:,1)./(Y(:,1)+Km_acylACP)./(1+Y(:,3).*Y(:,3).*Y(:,3)/Ki_ppGpp^3);

FAS_rate=FASm*Y(:,4).*acp./(acp+Km_ACP)*AcCoA/(AcCoA+Km_AcCoA)./(1+Y(:,1)/Ki_acylACP);

P1P2 = 1./(1+Y(:,3).*Y(:,3)/(ki_P1P2_ppGpp)^2);

VFA=Vtes*Y(:,1)./(Y(:,1)+Km_tes);

figure (1)

plot(T/60,Y(:,1)/20,'k');

hold on;

plot(T/60,Y(:,3)/1000,'r');

hold on;

plot(T/60,Y(:,5),'g');

hold on;

title('Acyl-ACP-black; ppGpp3-red; GFP-green');

figure (2)

plot(T/60,PL_rate/1000,'k');

hold on;

plot(T/60,FAS_rate/1000,'k:');

hold on;

plot(T/60,VFA*kfa/1000,'b');

hold on;

title('PLS-black (FAS-dot); FAflux-blue');

figure (3)

plot(T/60,Y(:,3)/1000,'r');

hold on;

%plot(t_Mur/60,ppGpp_Mur/1000,'LineStyle','non','Marker','diamond','MarkerEdgeColor','r','MarkerFaceColor','r','MarkerSize',6);

%hold on;

plot(T/60,Y(:,6),'g');

hold on;

plot(T/60,Y(:,9),'g:');

hold on;

plot(T/60,Y(:,4),'b');

hold on;

%plot(t_OD600_M/60,OD600_M,'b:');

%hold on;

plot(T/60,P1P2,'k');

hold on;

title('ppGpp-red; P1P2-black; rib-blue; nutr-green (lim-dot)');

figure (4)

plot(T/60,Y(:,8),'k');

hold on;

plot(t_OD_L,OD_l0,'LineStyle','non','Marker','^','MarkerEdgeColor','k','MarkerFaceColor','k','MarkerSize',6);

hold on;

plot(t_OD_L,OD_l1,'LineStyle','non','Marker','o','MarkerEdgeColor','k','MarkerFaceColor','k','MarkerSize',6);

hold on;

plot(t_OD_L,OD_l2,'LineStyle','non','Marker','s','MarkerEdgeColor','k','MarkerFaceColor','k','MarkerSize',6);

hold on;

plot(t_OD_L,OD_l3,'LineStyle','non','Marker','diamond','MarkerEdgeColor','k','MarkerFaceColor','k','MarkerSize',6);

hold on;

title('OD');

figure (5)

plot(T/60,Y(:,7),'g');

hold on;

plot(t_FA,FA_wt,'LineStyle','non','Marker','diamond','MarkerEdgeColor','k','MarkerFaceColor','k','MarkerSize',6);

hold on;

plot(t_FA,FA,'LineStyle','non','Marker','o','MarkerEdgeColor','k','MarkerFaceColor','k','MarkerSize',6);

hold on;

title('FA; line 0');

the program uses the following function (file named **Model_biosens_ppGpp.m**)

function Func = Model_biosens_ppGpp(t,y,ACPtot,Km_AcCoA,Km_ACP,Km_acylACP,FASm,VmPLS,AcCoA,kp_ppGpp,km_ppGpp,k0m_ppGpp,Ki_ppGpp,Ki_acylACP,knutr,ki_P1P2_ppGpp,kprib,kmrib,kI,kGFP,Ki_I,Km_tes,Vtes,Kgr,klim,Km_Ac_pp,V0,kfa);

Func = zeros(9, 1);

% y(1) Acyl-ACP

% y(2) I

% y(3) ppGpp

% y(4) rib

% y(5) GFP

% y(6) nutr

% y(7) FA

% y(8) N

% y(9) lim

P1P2=1/(1+(y(3)/ki_P1P2_ppGpp)^2);

ACP=ACPtot-y(1);

VFAS=FASm*y(4)*ACP/(ACP+Km_ACP)*AcCoA/(AcCoA+Km_AcCoA)/(1+y(1)/Ki_acylACP);

Vpls=VmPLS*y(4)*y(1)/(y(1)+Km_acylACP)/(1+(y(3)/Ki_ppGpp)^3);

vg=Kgr*y(6)*y(4)*Vpls/(Vpls+V0);

vFA=Vtes*y(1)/(y(1)+Km_tes);

Func(1) = VFAS-Vpls-vFA*kfa;

Func(2) = kI*(P1P2-y(2));

Func(3) = kp_ppGpp*y(4)-y(3)*(km_ppGpp*y(9)+k0m_ppGpp)*y(1)/ACP;

Func(3) = kp_ppGpp*y(4)-y(3)*(km_ppGpp*y(9)+k0m_ppGpp)*y(1)/(y(1)+Km_Ac_pp);

Func(4) = kprib*P1P2-kmrib*y(4);

Func(5) = kGFP*(1/(1+(y(2)/Ki_I))^4-y(5));

Func(6) = -knutr*y(8)*y(6)/(y(6)+0.001);

Func(7) = vFA*y(8)*y(6)/(y(6)+0.001);

Func(8) = y(8)*vg;

Func(9) = -klim*y(8)*y(9)/(y(9)+0.001);
